# 2,4-Dimethyl-6-nitro­aniline

**DOI:** 10.1107/S1600536812014547

**Published:** 2012-04-13

**Authors:** Hu-Kui Chen

**Affiliations:** a Baoji University of Arts and Sciences, Department of Chemistry, Baoji 721013, Shaanxi, People’s Republic of China

## Abstract

The asymmetric unit of the title compound, C_8_H_10_N_2_O_2_, contains two independent mol­ecules, which are linked by weak N—H⋯O hydrogen-bonding inter­actions between the amino and nitro groups. The independent molecules are both approximately planar with r.s.d. deviations of 0.0216 and 0.0161 Å.

## Related literature

For applications of the title compound and background to the synthesis, see: Qian (2005 ▶); Qi *et al.* (2009 ▶); Liang (2000 ▶); Hu *et al.* (2010 ▶).
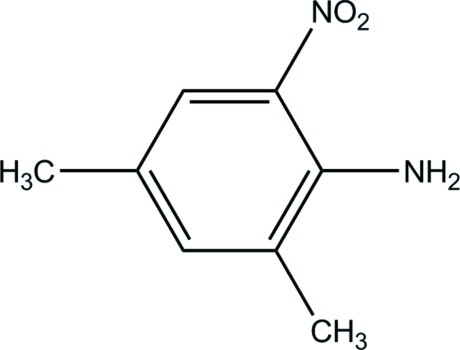


## Experimental

### 

#### Crystal data

C_8_H_10_N_2_O_2_
*M* *_r_* = 166.18
Monoclinic, 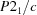

*a* = 6.997 (2) Å
*b* = 14.919 (4) Å
*c* = 15.907 (5) Å
β = 101.176 (4)°
*V* = 1629.1 (8) Å^3^
*Z* = 8
Mo *K*α radiation
μ = 0.10 mm^−1^
*T* = 163 K
0.37 × 0.35 × 0.24 mm

#### Data collection

Rigaku AFC10/Saturn724+ diffractometer
10540 measured reflections
4325 independent reflections
3104 reflections with *I* > 2σ(*I*)
*R*_int_ = 0.027

#### Refinement

*R*[*F* ^2^ > 2σ(*F* ^2^)] = 0.049
*wR*(*F* ^2^) = 0.134
*S* = 1.00
4325 reflections
237 parameters
H atoms treated by a mixture of independent and constrained refinement
Δρ_max_ = 0.32 e Å^−3^
Δρ_min_ = −0.21 e Å^−3^

### 

Data collection: *CrystalClear* (Rigaku/MSC, 2008) ▶; cell refinement: *CrystalClear*; data reduction: *CrystalClear*; program(s) used to solve structure: *SHELXS97* (Sheldrick, 2008 ▶); program(s) used to refine structure: *SHELXL97* (Sheldrick, 2008 ▶); molecular graphics: *SHELXTL* (Sheldrick, 2008 ▶); software used to prepare material for publication: *SHELXL97*.

## Supplementary Material

Crystal structure: contains datablock(s) I, global. DOI: 10.1107/S1600536812014547/zj2069sup1.cif

Structure factors: contains datablock(s) I. DOI: 10.1107/S1600536812014547/zj2069Isup2.hkl

Supplementary material file. DOI: 10.1107/S1600536812014547/zj2069Isup3.cml

Additional supplementary materials:  crystallographic information; 3D view; checkCIF report

## Figures and Tables

**Table 1:** Hydrogen-bond geometry (Å, °)

*D*—H⋯*A*	*D*—H	H⋯*A*	*D*⋯*A*	*D*—H⋯*A*
N1—H1*A*⋯O3	0.91 (2)	2.27 (2)	3.166 (2)	167.9 (18)
N3—H3*B*⋯O4	0.93 (2)	1.92 (2)	2.631 (2)	131.3 (17)
N3—H3*A*⋯O2^i^	0.89 (2)	2.30 (2)	3.1667 (19)	165.8 (17)
N1—H1*B*⋯O2	0.86 (2)	1.972 (18)	2.6233 (19)	131.4 (16)

Symmetry code: (i) 
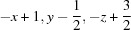
.

**Fig. 1.**
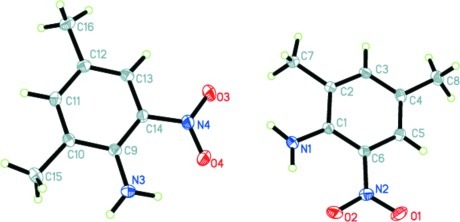
The molecular structure of (I), with the atom-numbering scheme. Displacement ellipsoids are drawn at the 30% probability level.

**Fig. 2.**
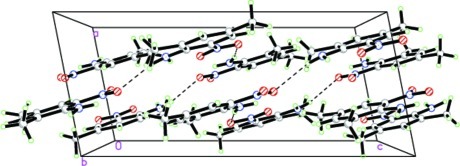
Part of the packing of the title compound, viewed down the b axis. Dashed lines indicate hydrogen bonds.

## supplementary crystallographic information

### Comment

The tittle compound,2,4-dimethyl-6-nitroaniline, is a very important aromatic organic intermediate, which can be utilized to synthesize dyes and pigment. It is practical significant to research and develop 2,4-dimethyl-6-nitroaniline because of difficult synthesis process, higher costs and bad yield. To improve the reaction condition and enlarge the needs of it, we report here the crystal structure of the title compound 2,4-dimethyl-6-nitroaniline,(I).

The molecular structure of (I) is shown in Fig.1. The asymmetric unit contains two title molecules of 2,4-dimethyl-6-nitroaniline. The non-hydrogen atoms of these molecules molecule are situated in a fair plane with r.m.s.deviation of 0.0216 Å and 0.0161 Å. The bond lengths and angles are within normal ranges in both molecules. In the crystal structure, the two molecules are not parallel but have a dihedral angle of 2.19 (0.02)°. The intermolecular N—H···O hydrogen bonds (Table 1) link the molecules (Fig. 2), in which they may be effective in the stabilization of the structure.

### Experimental

A solution of 2,4-dimethylaniline(24.2 g, 0.2 mol), acetic acid(23 ml) and acetic anhydride (19 ml) was refluxed for 1 h and cooled to 35°C. Then, the mixed acid of concentrated sulfuric acid(35 ml) and concentrated nitric acid (17 ml) was slowly dropped into it after concentrated sulfuric acids(40 ml) was added. The mixture was reacted for 1 h and cooled to the room tempreture, and added to the cooled water.The resultant white solid 2,4-dimethylacetanilide was filtered and washed with cooled water. 2,4-dimethylacetanilide was then was added to the solution of 70% sulfuric acids (80 ml) and refluxed for 1 h, and slowly added to the cooled water.Orange-red precipitate began to appear. The precipitate was filtered and washed with water until the pH value of the filtrate is 7. The solid product was collected after dried at 80 °C(yield 82.5%, mp.70–72 °C).The crystals of 2,4-dimethyl-6-nitroaniline suitable for X-ray analysis were obtained by dissolving (I) (0.1 g) in methanol (20 ml) and evaporating the solvent slowly at room temperature for about 10 d.

### Refinement

H atoms were positioned geometrically, with N—H = 0.86–0.93 Å (for NH) and C—H = 0.95 and 0.98 Å for aromatic and methyl H, respectively, and constrained to ride on their parent atoms, with *U*_iso_(H) = *xU*_eq_(C), where *x* = 1.2 for methyl and aromatic H.

### Crystal data

**Table:**

C_8_H_10_N_2_O_2_	*F*(000) = 704
*M**_r_* = 166.18	*D*_x_ = 1.355 Mg m^−^^3^
Monoclinic, *P*2_1_/*c*	Mo *K*α radiation, λ = 0.71073 Å
Hall symbol: -P 2ybc	Cell parameters from 4298 reflections
*a* = 6.997 (2) Å	θ = 2.6–29.1°
*b* = 14.919 (4) Å	µ = 0.10 mm^−^^1^
*c* = 15.907 (5) Å	*T* = 163 K
β = 101.176 (4)°	Block, red
*V* = 1629.1 (8) Å^3^	0.37 × 0.35 × 0.24 mm
*Z* = 8	

### Data collection

**Table:**

Rigaku AFC10/Saturn724+ diffractometer	3104 reflections with *I* > 2σ(*I*)
Radiation source: Rotating Anode	*R*_int_ = 0.027
Graphite monochromator	θ_max_ = 29.1°, θ_min_ = 2.6°
Detector resolution: 28.5714 pixels mm^-1^	*h* = −9→9
phi and ω scans	*k* = −20→13
10540 measured reflections	*l* = −19→21
4325 independent reflections	

### Refinement

**Table:**

Refinement on *F*^2^	Primary atom site location: structure-invariant direct methods
Least-squares matrix: full	Secondary atom site location: difference Fourier map
*R*[*F*^2^ > 2σ(*F*^2^)] = 0.049	Hydrogen site location: inferred from neighbouring sites
*wR*(*F*^2^) = 0.134	H atoms treated by a mixture of independent and constrained refinement
*S* = 1.00	*w* = 1/[σ^2^(*F*_o_^2^) + (0.0669*P*)^2^ + 0.169*P*] where *P* = (*F*_o_^2^ + 2*F*_c_^2^)/3
4325 reflections	(Δ/σ)_max_ < 0.001
237 parameters	Δρ_max_ = 0.32 e Å^−^^3^
0 restraints	Δρ_min_ = −0.21 e Å^−^^3^

### Special details

**Table:**

Geometry. All e.s.d.'s (except the e.s.d. in the dihedral angle between two l.s. planes) are estimated using the full covariance matrix. The cell e.s.d.'s are taken into account individually in the estimation of e.s.d.'s in distances, angles and torsion angles; correlations between e.s.d.'s in cell parameters are only used when they are defined by crystal symmetry. An approximate (isotropic) treatment of cell e.s.d.'s is used for estimating e.s.d.'s involving l.s. planes.
Refinement. Refinement of *F*^2^ against ALL reflections. The weighted *R*-factor *wR* and goodness of fit *S* are based on *F*^2^, conventional *R*-factors *R* are based on *F*, with *F* set to zero for negative *F*^2^. The threshold expression of *F*^2^ > σ(*F*^2^) is used only for calculating *R*-factors(gt) *etc*. and is not relevant to the choice of reflections for refinement. *R*-factors based on *F*^2^ are statistically about twice as large as those based on *F*, and *R*- factors based on ALL data will be even larger.

### Fractional atomic coordinates and isotropic or equivalent isotropic displacement parameters (Å^2^)

**Table:**

	*x*	*y*	*z*	*U*_iso_*/*U*_eq_	
O1	0.45092 (17)	0.90397 (8)	0.58620 (8)	0.0475 (3)	
O2	0.44448 (18)	0.76422 (8)	0.61993 (7)	0.0461 (3)	
O3	0.1904 (2)	0.44185 (8)	0.46076 (8)	0.0515 (3)	
O4	0.28094 (19)	0.46393 (7)	0.59626 (8)	0.0488 (3)	
N1	0.32759 (19)	0.64184 (9)	0.50283 (9)	0.0337 (3)	
N2	0.42024 (17)	0.82485 (8)	0.56491 (8)	0.0331 (3)	
N3	0.29546 (19)	0.33074 (9)	0.70576 (9)	0.0349 (3)	
N4	0.22429 (19)	0.41310 (8)	0.53454 (9)	0.0351 (3)	
C1	0.31439 (18)	0.71292 (9)	0.44965 (9)	0.0253 (3)	
C2	0.25692 (19)	0.69862 (9)	0.35968 (9)	0.0267 (3)	
C3	0.2419 (2)	0.77019 (9)	0.30489 (10)	0.0293 (3)	
H3	0.2037	0.7591	0.2452	0.035*	
C4	0.28002 (19)	0.85946 (9)	0.33221 (10)	0.0294 (3)	
C5	0.33876 (19)	0.87389 (9)	0.41793 (10)	0.0278 (3)	
H5	0.3681	0.9331	0.4384	0.033*	
C6	0.35645 (18)	0.80271 (9)	0.47637 (9)	0.0251 (3)	
C7	0.2177 (2)	0.60435 (10)	0.32704 (11)	0.0385 (4)	
H7A	0.1968	0.6043	0.2643	0.046*	
H7B	0.1013	0.5812	0.3453	0.046*	
H7C	0.3296	0.5662	0.3502	0.046*	
C8	0.2570 (2)	0.93525 (10)	0.26839 (11)	0.0409 (4)	
H8A	0.1247	0.9595	0.2610	0.049*	
H8B	0.2800	0.9129	0.2133	0.049*	
H8C	0.3514	0.9826	0.2894	0.049*	
C9	0.24075 (18)	0.28267 (9)	0.63273 (9)	0.0258 (3)	
C10	0.21597 (19)	0.18803 (9)	0.63927 (10)	0.0281 (3)	
C11	0.15958 (19)	0.13826 (9)	0.56628 (10)	0.0306 (3)	
H11	0.1458	0.0753	0.5721	0.037*	
C12	0.12098 (19)	0.17490 (9)	0.48338 (10)	0.0293 (3)	
C13	0.14163 (19)	0.26569 (9)	0.47631 (9)	0.0283 (3)	
H13	0.1161	0.2930	0.4213	0.034*	
C14	0.20012 (19)	0.31878 (9)	0.54949 (9)	0.0262 (3)	
C15	0.2515 (2)	0.14538 (10)	0.72614 (11)	0.0394 (4)	
H15A	0.2234	0.0811	0.7203	0.047*	
H15B	0.1664	0.1730	0.7610	0.047*	
H15C	0.3879	0.1541	0.7540	0.047*	
C16	0.0600 (2)	0.11639 (11)	0.40631 (11)	0.0405 (4)	
H16A	−0.0791	0.1027	0.3993	0.049*	
H16B	0.1351	0.0605	0.4140	0.049*	
H16C	0.0843	0.1477	0.3552	0.049*	
H1A	0.284 (3)	0.5869 (14)	0.4823 (13)	0.056 (6)*	
H3B	0.325 (3)	0.3907 (14)	0.6975 (13)	0.066 (6)*	
H3A	0.350 (3)	0.3063 (13)	0.7554 (14)	0.057 (6)*	
H1B	0.355 (3)	0.6534 (11)	0.5569 (13)	0.043 (5)*	

### Atomic displacement parameters (Å^2^)

**Table:**

	*U*^11^	*U*^22^	*U*^33^	*U*^12^	*U*^13^	*U*^23^
O1	0.0653 (8)	0.0384 (6)	0.0380 (7)	−0.0122 (5)	0.0081 (6)	−0.0142 (5)
O2	0.0680 (8)	0.0467 (7)	0.0214 (6)	−0.0050 (5)	0.0030 (5)	0.0041 (5)
O3	0.0844 (9)	0.0360 (6)	0.0324 (7)	−0.0024 (6)	0.0068 (6)	0.0128 (5)
O4	0.0798 (9)	0.0277 (5)	0.0375 (7)	−0.0049 (5)	0.0080 (6)	−0.0050 (5)
N1	0.0471 (7)	0.0276 (6)	0.0246 (7)	−0.0015 (5)	0.0023 (6)	0.0057 (6)
N2	0.0349 (7)	0.0365 (7)	0.0277 (7)	−0.0036 (5)	0.0054 (5)	−0.0026 (6)
N3	0.0479 (8)	0.0333 (7)	0.0230 (7)	0.0004 (5)	0.0063 (6)	−0.0028 (6)
N4	0.0470 (7)	0.0285 (6)	0.0302 (7)	0.0015 (5)	0.0083 (5)	0.0037 (6)
C1	0.0249 (6)	0.0259 (6)	0.0251 (7)	0.0004 (5)	0.0045 (5)	0.0042 (5)
C2	0.0282 (7)	0.0262 (6)	0.0246 (8)	0.0007 (5)	0.0020 (5)	0.0003 (6)
C3	0.0325 (7)	0.0324 (7)	0.0217 (7)	0.0028 (5)	0.0019 (5)	0.0022 (6)
C4	0.0314 (7)	0.0277 (7)	0.0302 (8)	0.0013 (5)	0.0087 (5)	0.0069 (6)
C5	0.0292 (7)	0.0243 (6)	0.0308 (8)	−0.0013 (5)	0.0077 (5)	0.0005 (6)
C6	0.0260 (6)	0.0279 (7)	0.0217 (7)	−0.0014 (5)	0.0052 (5)	−0.0004 (6)
C7	0.0528 (9)	0.0293 (8)	0.0293 (9)	−0.0007 (6)	−0.0025 (7)	−0.0020 (6)
C8	0.0516 (9)	0.0351 (8)	0.0360 (10)	0.0033 (6)	0.0083 (7)	0.0126 (7)
C9	0.0279 (7)	0.0286 (7)	0.0220 (7)	0.0011 (5)	0.0075 (5)	−0.0008 (6)
C10	0.0295 (7)	0.0295 (7)	0.0264 (8)	0.0010 (5)	0.0081 (5)	0.0044 (6)
C11	0.0321 (7)	0.0256 (7)	0.0348 (9)	−0.0029 (5)	0.0079 (6)	0.0019 (6)
C12	0.0271 (7)	0.0318 (7)	0.0292 (8)	−0.0007 (5)	0.0058 (5)	−0.0038 (6)
C13	0.0295 (7)	0.0333 (7)	0.0226 (7)	0.0016 (5)	0.0062 (5)	0.0013 (6)
C14	0.0303 (7)	0.0235 (6)	0.0256 (7)	0.0013 (5)	0.0075 (5)	0.0012 (6)
C15	0.0476 (9)	0.0382 (8)	0.0325 (9)	0.0002 (6)	0.0082 (7)	0.0106 (7)
C16	0.0447 (9)	0.0397 (8)	0.0354 (10)	−0.0047 (6)	0.0032 (7)	−0.0106 (7)

### Geometric parameters (Å, º)

**Table:**

O1—N2	1.2353 (16)	C7—H7A	0.9800
O2—N2	1.2472 (17)	C7—H7B	0.9800
O3—N4	1.2288 (17)	C7—H7C	0.9800
O4—N4	1.2431 (17)	C8—H8A	0.9800
N1—C1	1.3484 (18)	C8—H8B	0.9800
N1—H1A	0.91 (2)	C8—H8C	0.9800
N1—H1B	0.86 (2)	C9—C14	1.407 (2)
N2—C6	1.4311 (19)	C9—C10	1.4287 (19)
N3—C9	1.3557 (19)	C10—C11	1.370 (2)
N3—H3B	0.93 (2)	C10—C15	1.498 (2)
N3—H3A	0.89 (2)	C11—C12	1.405 (2)
N4—C14	1.4424 (18)	C11—H11	0.9500
C1—C6	1.4189 (18)	C12—C13	1.369 (2)
C1—C2	1.426 (2)	C12—C16	1.498 (2)
C2—C3	1.3693 (19)	C13—C14	1.402 (2)
C2—C7	1.5058 (19)	C13—H13	0.9500
C3—C4	1.410 (2)	C15—H15A	0.9800
C3—H3	0.9500	C15—H15B	0.9800
C4—C5	1.363 (2)	C15—H15C	0.9800
C4—C8	1.507 (2)	C16—H16A	0.9800
C5—C6	1.4009 (19)	C16—H16B	0.9800
C5—H5	0.9500	C16—H16C	0.9800
			
C1—N1—H1A	120.3 (13)	C4—C8—H8A	109.5
C1—N1—H1B	116.4 (12)	C4—C8—H8B	109.5
H1A—N1—H1B	122.1 (17)	H8A—C8—H8B	109.5
O1—N2—O2	120.51 (13)	C4—C8—H8C	109.5
O1—N2—C6	119.66 (13)	H8A—C8—H8C	109.5
O2—N2—C6	119.83 (12)	H8B—C8—H8C	109.5
C9—N3—H3B	114.8 (13)	N3—C9—C14	125.17 (13)
C9—N3—H3A	123.1 (13)	N3—C9—C10	118.59 (13)
H3B—N3—H3A	116.9 (18)	C14—C9—C10	116.22 (13)
O3—N4—O4	120.95 (13)	C11—C10—C9	119.53 (13)
O3—N4—C14	119.40 (13)	C11—C10—C15	121.37 (13)
O4—N4—C14	119.64 (13)	C9—C10—C15	119.09 (13)
N1—C1—C6	124.71 (14)	C10—C11—C12	123.78 (13)
N1—C1—C2	118.94 (13)	C10—C11—H11	118.1
C6—C1—C2	116.34 (12)	C12—C11—H11	118.1
C3—C2—C1	119.62 (13)	C13—C12—C11	117.26 (13)
C3—C2—C7	121.49 (14)	C13—C12—C16	121.80 (14)
C1—C2—C7	118.88 (13)	C11—C12—C16	120.93 (13)
C2—C3—C4	123.61 (14)	C12—C13—C14	120.61 (14)
C2—C3—H3	118.2	C12—C13—H13	119.7
C4—C3—H3	118.2	C14—C13—H13	119.7
C5—C4—C3	117.38 (13)	C13—C14—C9	122.58 (13)
C5—C4—C8	121.84 (13)	C13—C14—N4	116.04 (13)
C3—C4—C8	120.78 (14)	C9—C14—N4	121.33 (13)
C4—C5—C6	120.99 (13)	C10—C15—H15A	109.5
C4—C5—H5	119.5	C10—C15—H15B	109.5
C6—C5—H5	119.5	H15A—C15—H15B	109.5
C5—C6—C1	122.03 (13)	C10—C15—H15C	109.5
C5—C6—N2	116.66 (12)	H15A—C15—H15C	109.5
C1—C6—N2	121.31 (12)	H15B—C15—H15C	109.5
C2—C7—H7A	109.5	C12—C16—H16A	109.5
C2—C7—H7B	109.5	C12—C16—H16B	109.5
H7A—C7—H7B	109.5	H16A—C16—H16B	109.5
C2—C7—H7C	109.5	C12—C16—H16C	109.5
H7A—C7—H7C	109.5	H16A—C16—H16C	109.5
H7B—C7—H7C	109.5	H16B—C16—H16C	109.5
			
N1—C1—C2—C3	179.68 (12)	N3—C9—C10—C11	179.60 (13)
C6—C1—C2—C3	−1.15 (19)	C14—C9—C10—C11	1.34 (19)
N1—C1—C2—C7	−1.48 (19)	N3—C9—C10—C15	−0.11 (19)
C6—C1—C2—C7	177.69 (12)	C14—C9—C10—C15	−178.37 (12)
C1—C2—C3—C4	−0.2 (2)	C9—C10—C11—C12	−0.9 (2)
C7—C2—C3—C4	−178.98 (14)	C15—C10—C11—C12	178.82 (13)
C2—C3—C4—C5	1.4 (2)	C10—C11—C12—C13	0.0 (2)
C2—C3—C4—C8	−178.87 (13)	C10—C11—C12—C16	179.93 (13)
C3—C4—C5—C6	−1.3 (2)	C11—C12—C13—C14	0.36 (19)
C8—C4—C5—C6	179.02 (13)	C16—C12—C13—C14	−179.57 (13)
C4—C5—C6—C1	−0.1 (2)	C12—C13—C14—C9	0.2 (2)
C4—C5—C6—N2	179.65 (12)	C12—C13—C14—N4	177.77 (12)
N1—C1—C6—C5	−179.59 (13)	N3—C9—C14—C13	−179.16 (13)
C2—C1—C6—C5	1.29 (19)	C10—C9—C14—C13	−1.03 (19)
N1—C1—C6—N2	0.7 (2)	N3—C9—C14—N4	3.4 (2)
C2—C1—C6—N2	−178.42 (12)	C10—C9—C14—N4	−178.49 (12)
O1—N2—C6—C5	2.09 (19)	O3—N4—C14—C13	1.16 (19)
O2—N2—C6—C5	−177.89 (12)	O4—N4—C14—C13	−178.08 (13)
O1—N2—C6—C1	−178.19 (12)	O3—N4—C14—C9	178.78 (14)
O2—N2—C6—C1	1.8 (2)	O4—N4—C14—C9	−0.5 (2)

### Hydrogen-bond geometry (Å, º)

**Table:**

*D*—H···*A*	*D*—H	H···*A*	*D*···*A*	*D*—H···*A*
N1—H1*A*···O3	0.91 (2)	2.27 (2)	3.166 (2)	167.9 (18)
N3—H3*B*···O4	0.93 (2)	1.92 (2)	2.631 (2)	131.3 (17)
N3—H3*A*···O2^i^	0.89 (2)	2.30 (2)	3.1667 (19)	165.8 (17)
N1—H1*B*···O2	0.86 (2)	1.972 (18)	2.6233 (19)	131.4 (16)

Symmetry code: (i) −*x*+1, *y*−1/2, −*z*+3/2.
